# COVID-19 Result Follow-Up Process in the Pediatric Emergency Department Setting

**DOI:** 10.1017/dmp.2020.403

**Published:** 2020-10-22

**Authors:** Shaina Newman, Joelle Simpson, Ashley Perritt, Sephora Morrison, Deena Berkowitz, Kathleen Brown, Tress Goodwin

**Affiliations:** Children’s National Medical Center, Washington, DC, USA

**Keywords:** COVID-19, delivery of health care, emergency medicine, disease outbreaks, emergency preparedness

## Abstract

The novel coronavirus disease 2019 (COVID-19) pandemic upended the world. As emergency departments and hospitals across the nation and world braced themselves for the surge of this new disease, the emergency department (ED) at Children’s National Hospital (CNH) quickly created a process to address surges in patient visits and follow-ups for coronavirus testing. Within 2 wk of the first reported pediatric patient diagnosed with COVID-19 in the Washington, DC, metropolitan area, CNH ED implemented a new comprehensive follow-up process. This article describes the novel process that ensured timely notification of testing results, enabled patients to speak remotely with ED providers, increased patient and staff safety by reducing unnecessary exposures, and suggested a good patient experience. With over 1900 patients discharged pending their COVID-19 results, the program is successful. We anticipate expansion into antibody testing and notification as the pandemic progresses.

Children’s National Hospital (CNH) is a large, urban, Level 1 trauma center and tertiary care children’s hospital with more than 100,000 pediatric emergency department (ED) visits annually. ED medical providers include board-certified pediatric emergency medicine physicians, general pediatricians, pediatric residents, and advanced practice providers (APP), including physician assistants and nurse practitioners.

COVID-19 was first diagnosed in the Washington, DC, region in early March of 2020.^1^ Despite early reports claiming lower infection rates, and morbidity in pediatric patients, CNH ED prepared for a potential surge.^2,3^ Similar to other EDs across the world, CNH experienced reductions in patient volumes due to fear of contracting COVID, but we also did see an increase in requests for COVID-19 testing.^4,5^ The testing in our ED was done by means of nasopharyngeal swabs obtained by a provider or nurse who was wearing full PPE, including an N95, face shield, gown, and gloves in a closed-off room. Our hospital offers an in-house COVID-19 polymerase chain reaction (PCR) test with an average turnaround time of approximately 4-6 h for results. Patients who were clinically stable and unlikely to need admission were offered a phone follow-up for results, which was done by providers from home. This reduced staff and patient exposure, and was in line with other hospitals who have reallocated staff to new tasks to reduce exposure and maintain a healthy workforce.^6^

## Follow-Up Process

**Figure 1.**
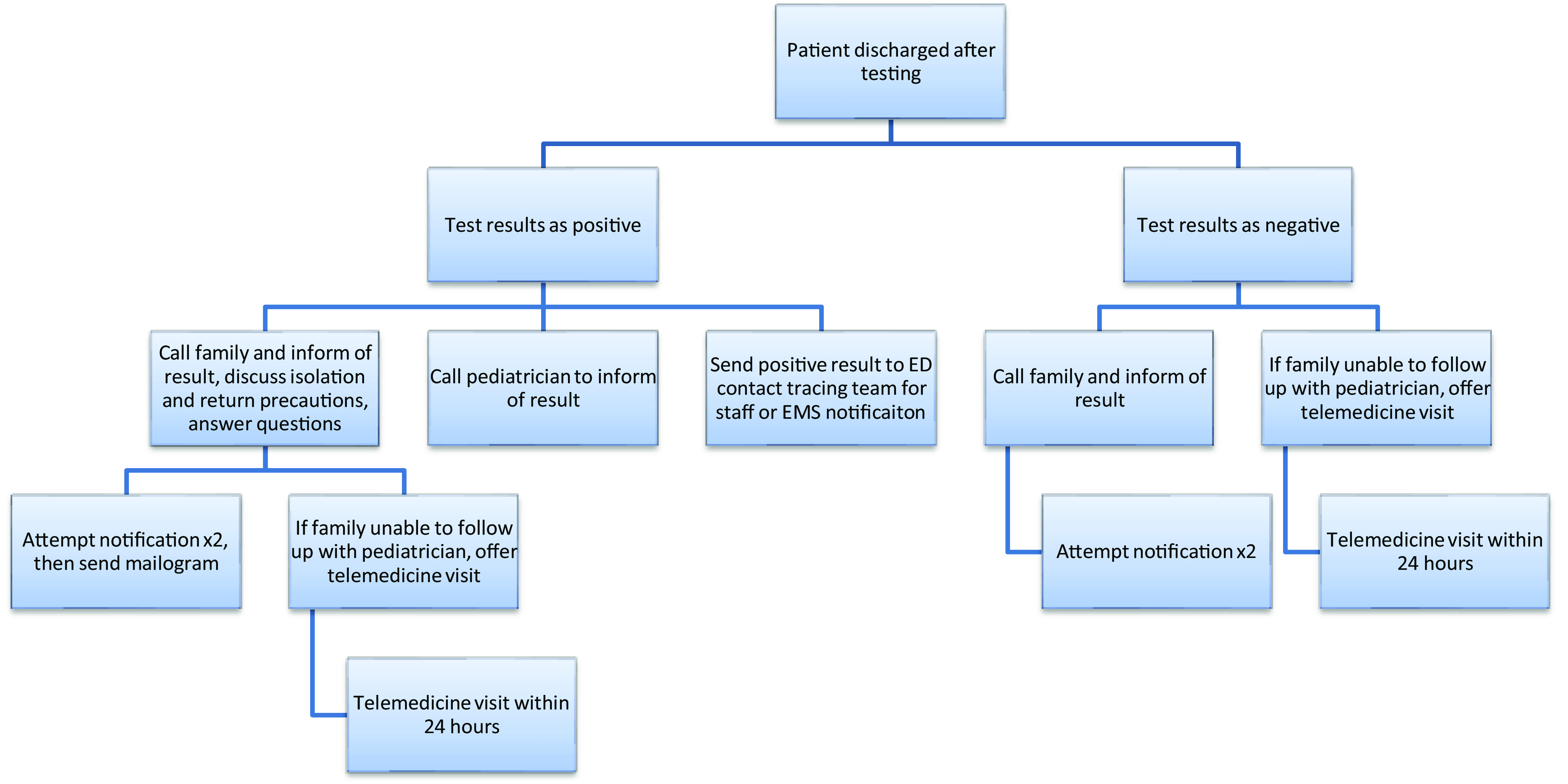
Flow chart of the follow-up process.

Our follow-up process is multifaceted and includes (1) comprehensive discharge instructions, (2) a phone call to discuss results from an ED APP, primary care provider (PCP) notification, and (3) the option of a telemedicine follow-up with a pediatrician (Figure 1).

### Discharge Instructions

Patients who were clinically stable but awaiting COVID-19 PCR results were offered the option to be discharged home and logged for phone follow-up to share results within 24 h. All patients and their families were instructed to quarantine until they received a call from the ED. A new discharge instruction form was created for these families that outlined steps to quarantine, provided anticipatory guidance about the course of illness for COVID-19 and other differential diagnoses, as well as return precautions. This form was also translated into Spanish as a resource for our large Spanish speaking population.

To troubleshoot challenges of providers forgetting to place accurate and current phone numbers for families, we worked with our information technology (IT) department to create a “hard stop” into the electronic medical record, requiring the ordering provider to ensure up-to-date contact information was entered before a COVID-19 test could be ordered.

### Staffing

To staff the newly added COVID-19 results callback system, the ED reconfigured our current staffing hours. Due to our decrease in volumes, and particularly decrease in the volume of low acuity patients, we transitioned 1 APP shift typically in the low acuity area to be solely focused on callbacks. The APP worked from home to make the calls, using the online electronic health record (EHR). Similarly, physicians staffed in low acuity shifts shifted to fill the telemedicine position also working remotely from home.

### Follow-Up With Patient and PCP

The follow-up team of APPs contacted all patients discharged from the ED with COVID-19 test results pending. All patients were attempted phone notification 2 times. If a patient who tested positive was not reachable after 2 attempts over a 2-d period, a mailgram by means of certified courier was sent to their house to ensure they were notified.

**Figure 2.**
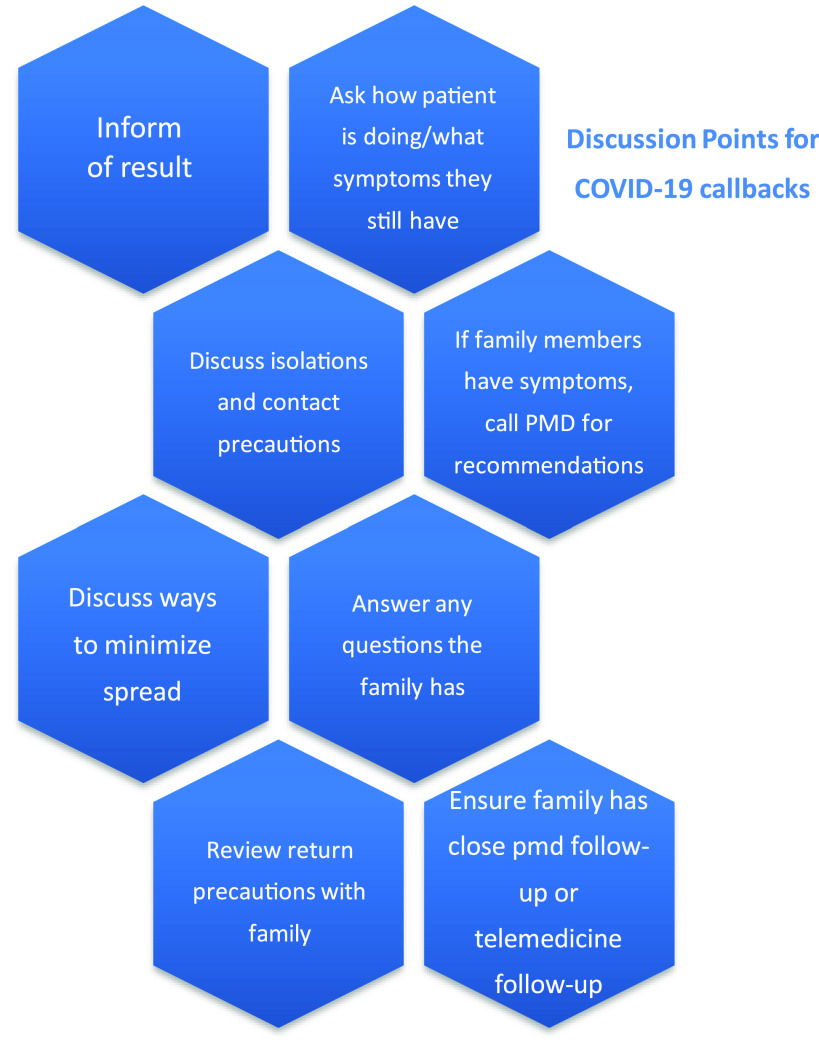
Discussion points for COVID-19 callbacks.

For patients testing positive, we developed a set of discussion points for each APP to review with families to ensure that all families received consistent information (Figure 2). These points included isolation precautions, what to do if a family member develops symptoms, return precautions, ways to minimize spread, and anticipatory guidance. APPs also answered any additional questions parents had about COVID and caring for their child. All PCPs of patients testing positive received calls from APPs to inform of results. The central lab in our hospital made all necessary notifications to the Washington, DC, Department of Health.

### Telemedicine Follow-Up

Early on in the follow-up process, we found some patients and families had questions that could not be answered by the APP by means of a brief phone call. And while we encouraged follow-up with their own PCP, many did not have a PCP or had difficulties in scheduling an appointment. In response, the ED urgently launched a telemedicine follow-up program in April 2020. Parents called with COVID-19 results, either negative or positive, were offered telemedicine follow-up with an ED provider if they were identified as unable to contact their PCP. The appointments were arranged through our scheduling department, and the telemedicine provider used a secure ZOOM platform to conduct the appointments.

### Contact Tracing

The new COVID-19 follow-up process facilitated contact tracing within the department. Early on in the pandemic, the hospital had a policy of contacting all staff members with exposure to a positive patient in the ED. The follow-up APP collected medical record numbers of all of the positive patients and send them to the infection control and occupational health teams, who would then identify staff and determine their risk. Once universal precautions were widely accepted and our rate of staff infection remained very low, this process was discontinued.

## Results

**Figure 3.**
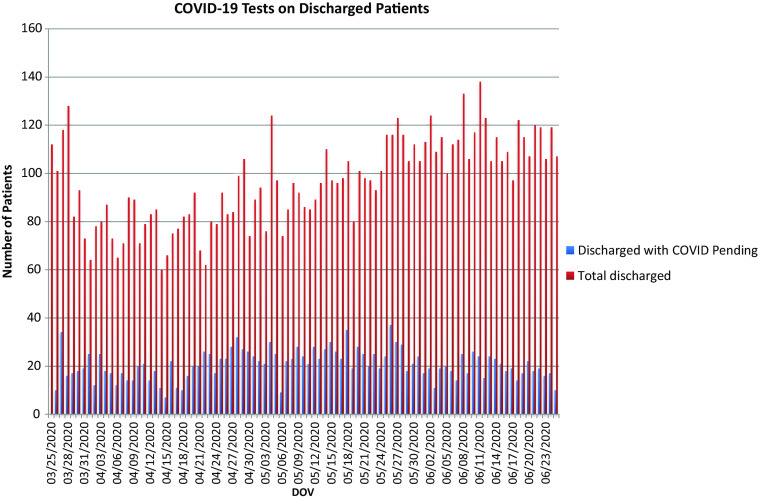
COVID-19 tests on discharged patients.

From March 25 to June 25, CNH ED completed 3329 COVID-19 PCR tests, with 353 positives (10.6%). A total of 1908 of the 3329 patients who received a COVID-19 test were discharged home pending their results, including 277 of the 353 positive patients (78%) (Figure 3).

**Figure 4.**
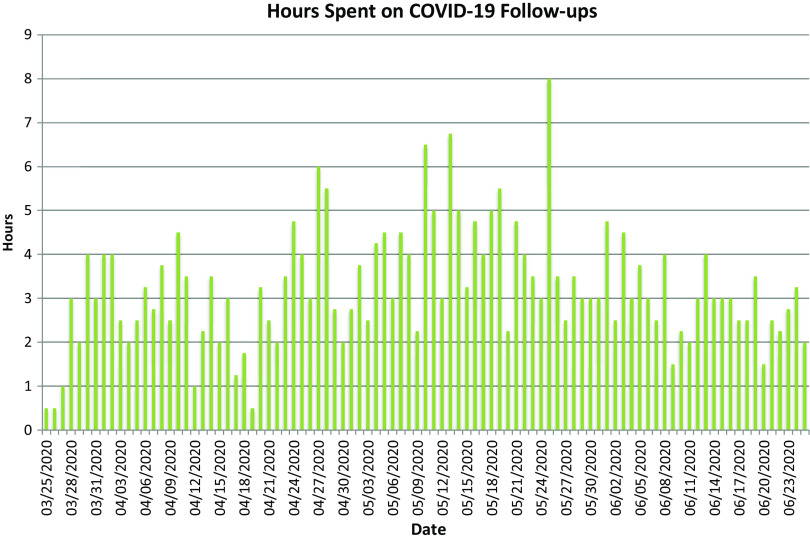
Hours spent on COVID-19 follow-ups.

In the CNH ED, 1 APP per day is allotted 3 h of general follow-up time for labs, radiology over reads, and family calls. Due to the surge of COVID-19 testing, a separate APP was assigned to do all COVID-19 follow-up calls. APPs spent an average of 3.3 h per day on COVID-19 follow-up calls, with a range of 0.5-8 h per day (Figure 4). A total of 90% reported spending 5-15 min on the phone with families who had positive COVID-19 tests, and 90% reported 0-5 min needed for reporting negative results. The remaining 10% of APPs spent greater than 15 and 5 min, respectively. The follow-up APPs reached patients on the first call more than 50% of the time. Five certified letters had to be sent to patients with positive results that we were unable to reach by means of telephone.

The most common question when reporting positive results was if additional family members required testing. Other common questions included what medications to give their children, how long to quarantine, and if parents or an older patient could return to work. These common questions led to the development for an “FAQ” (frequently asked questions) document addressing these concerns (Supplemental Appendix 1, which is available online). The APP placed a note in the patient’s chart in the EHR, documenting the successful call and transmission of results. In addition, if the patient requested a telemedicine appointment, their information was sent to the department scheduler to initiate the appointment.

In the 3-mo period from March 25 to June 25, 183 telemedicine appointments were scheduled, with 88.5% of these patients completing their visit (21 no-show visits). Telemedicine appointments were offered Monday through Saturday in 30-min time slots.

We cross-referenced patients who had COVID-19 testing in the ED with our patient experience feedback surveys. Of the 3586 patients who were tested for COVID-19, 723 (20%) responded to the survey. Of those, 141 (21%) added additional comments and had a COVID-19 PCR follow-up call that was documented in the EHR about their COVID-19 results. One comment directly addressed the follow-up callback process and disagreed with advice provided. Overall, 77 (55%) of the comments received were positive (Table 1).

**Table 1.** Comments received

Comment Type	Comment Count (%)
Mixed	21 (15%)
Negative	38 (27%)
Neutral	5 (3%)
Positive	77 (55%)
Grand total	141 (100%)

## Discussion

The COVID-19 follow-up process developed at CNH ED enabled quick discharge of stable patients with pending COVID-19 tests, ensured timely and comprehensive follow-up to our patients, and reallocated staffing hours not required due to decreased patient volumes.

For our patients and their families, this newly developed process provided numerous benefits. From the initial ED visit, having a follow-up process allowed them to spend less time in the ED and, thus, decrease their risk of exposure. This also allowed families to have a dedicated callback to answer any questions they had, and the option of an additional telemedicine follow-up from an ED physician, all from the safety of their own home. For those tested and providing feedback on their patient experience by means of comment responses to our survey, the majority were noted to be positive. However, due to the lack of direct commentary on the follow-up process, it is difficult to attribute these positive responses to the follow-up process provided.

For our ED staff, the rapid discharge of stable patients also decreased prolonged exposure to COVID-19, as lengthy exposure has been shown to increase risk of contracting the disease.^7^ In fact, the vast majority of patients who tested positive at our institution were discharged safely home. By enabling staff to work hours from home on callbacks and telemedicine appointments, it allowed staff to fulfill work hours while reducing exposure to COVID-19. This is of the utmost importance because it protected our workforce and likely also reduced the asymptomatic spread of infection by having providers work remotely.^5^

This follow-up process is broadly applicable to any health-care organization, including the public health sector, urgent cares, other EDs, as well as outpatient offices. As the COVID-19 pandemic evolves, this process can be adapted for other uses, including antibody testing, COVID-19 and other vaccination programs, and possible subsequent vaccine reactions. In addition, as ED volumes decreased, we transitioned staff hours to accommodate the expanded need for follow-ups and telemedicine shifts, in alignment with other hospitals that reallocated staff due to an increasing demand for telemedicine during the COVID-19 pandemic.^6^ As we move forward, we hope to structure similar programs dependent on the need and expand to meet additional health-care needs. Using a home follow-up process for other results, such as strep and urine studies, can reduce ED length of stays, yet still ensure appropriate care. In addition, a remote follow-up can be considered for situations that would typically require return visits, such as wound checks and patient and family perceived worsening disease course. Expanding and improving the follow-up process ensures our patients and families receive excellent care while minimizing time spent in the ED and reducing the need for return visits.

## Supplementary material

For supplementary material accompanying this paper visit https://doi.org/10.1017/dmp.2020.403. click here to view supplementary material
