# Supplementary material for: COVID-19 Result Follow-Up Process in the Pediatric Emergency Department Setting
Source: Disaster Med Public Health Prep. 2020 Oct 22:1–5. doi: 10.1017/dmp.2020.403 (PMC7884666; doi:10.1017/dmp.2020.403)
Supplement: Supplementary file 1 [file S1935789320004036sup001.docx]

**Appendix 1**

**Information from the CDC website. Please refer to website or refer families to website if needed**

**Patient tested positive, how do we isolate?**

- Patient that is positive needs to stay in isolation until:
  - - **3 days with no fever AND**
      - respiratory symptoms have improved AND
        - 10 days since symptoms first appeared
    - If pt tested positive and was asymptomatic they must isolate for 10 days since the test was done
      - If at any point the patient becomes symptomatic then refer to the symptomatic recommendations for length of isolation above

**When should we return to the ER?**

- Trouble breathing
- Persistent pain or pressure in the chest
- New confusion or inability to arouse
- Bluish lips or face
- Dehydration
- Any other concerns for child’s health

**What can I do to prevent my families from catching/spreading coronavirus?**

- If possible, assign ONE primary caregiver to the child who is sick.
  - Who should be the primary caregiver:
    - If possible avoid having the primary caregiver be someone who has chronic medical illnesses, i.e. diabetes, asthma, copd, hypertension, history of immunosuppression, cancer patient, etc.
    - The primary caregiver should not be going to work until both the child and themselves are well
  - This caregiver and the child should (if possible)
    - Have their own section of the house that the other household members do not use
    - Have their own bathroom that other household member do not use
    - The other household members can drop off food/medicines/etc.
    - All household members should avoid leaving the house, but if necessary the people leaving the house should be the other household members, NOT the primary caregiver for the sick child
    - The primary caregiver should try to wear a mask even though they are providing close contact care, this can offer some protection
- Further general recommendations for the non-primary care giver of the sick child:
  - Avoid public transportation and ride share/taxis
  - Clean “high touch” surfaces every day- ie counters, tables, doors, bathroom fixtures, keyboards, tablets, bedside tables
  - Wear masks in the house when you cannot maintain 6ft of distancing from others, especially when in common spaces i.e. hallways, bathrooms that may have to be shared by sick patient and primary caregiver
  - Wear masks in public spaces where you cannot maintain 6ft distance
  - Remember that those household members that are elderly, have chronic medical conditions should take EXTRA precautions including masks, avoid any contact with sick patient or primary caregiver, staying somewhere else until all household members are well.
- Recommendations for all individuals:
  - Wash your hands for 20 seconds with soap and water, or use a hand sanitizer that contains 60% alcohol or more
  - Don’t touch your eyes, nose or mouth
  - Cover your mouth and nose with a tissue when you cough or sneeze, and immediately throw away tissue, wash hands

**Should family members be tested?**

- There is no recommendation for testing of asymptomatic people. Close contacts should monitor their health; they should call their healthcare provider right away if they develop symptoms suggestive of COVID-19 (e.g., fever, cough, shortness of breath)

**What to do about work?**

- Those living in the same household as this positive COVID-19 patient should contact their employers to inform them of the exposure
  - If possible all household members should quarantine
    - Quarantine means stay at home, minimize exposure to the community and try to stay 6ft away from positive patient

**What to do about church/social events?**

- No large gatherings or any social gatherings until sick patient and primary caregiver are clear of symptoms as described above
  - Unless one of these individuals requires medical care they should be at home, in isolation and other household members should be providing them with food/drinks/medicines
- For other family members, they should avoid any social gatherings until the entire household is well again
  - Avoid any situations where you can be within 6 feet of others

**If my child is negative:**

- While reassuring news, given tests are not 100% accurate we still recommend limiting social exposure until child and household is feeling better without symptoms suspicious of COVID-19 including fever, cough, congestion, stomach problems, etc. All pts should f/u with pcp or telehealth regardless of test results
